# Methyl 5,7-dihydr­oxy-2,2,9-trimethyl-6,11-dioxo-6,11-dihydro-2*H*-anthra[2,3-*b*]pyran-8-carboxyl­ate

**DOI:** 10.1107/S160053680803794X

**Published:** 2008-11-22

**Authors:** Diderot Noungoue Tchamo, Lydia Brelot, Cyril Antheaume, Silvère Ngouela, Annelise Lobstein

**Affiliations:** a Department of Organic Chemistry, Faculty of Science, University of Yaoundé I, P.O. Box 812 Yaoundé, Cameroon; b Institut de Chimie de Strasbourg, Université Louis Pasteur, Service de Radiocristallographie, UMR 7177 CNRS, 4 rue Blaise Pascal, 67070 Strasbourg Cedex, France; c Laboratory of NMR, LC1 IFR85, University Louis Pasteur, Faculty of Pharmacy, Strasbourg, France; d Laboratory of Pharmacognosy, LC1 UMR-CNRS 7175, University Louis Pasteur, Faculty of Pharmacy, Strasbourg, France

## Abstract

The title compound, C_22_H_18_O_7_, also known as laurentiquinone B, is a new anthraquinone which was isolated from *Vismia laurentii*, a Cameroonian medicinal plant. The asymmetric unit contains two independent mol­ecules. Each of them contains four fused rings, three of which are coplanar and typical of anthracene, while the heterocyclic rings adopt envelope conformations. Intra­molecular O—H⋯O hydrogen bonds result in the formation of two planar rings, which are also almost coplanar with the adjacent rings. In the crystal structure, inter­molecular O—H⋯O and C—H⋯O hydrogen bonds link the mol­ecules and a π–π contact is also present [centroid-centroid distance = 3.967 (3) Å].

## Related literature

For the biosynthesis of anthraquinones, see: Birch *et al.* (1965 ▶); Shibata & Ikekawa (1963 ▶). For the bioactivity of anthraquinones, see: ; Adwankar & Chitnis (1982 ▶); Sittie *et al.* (1999 ▶); Rath *et al.* (1995 ▶); Ismail *et al.* (1997 ▶); Nagem & de Oliveira (1997 ▶); Nguemeving *et al.* (2006 ▶). For the pharmacology of *Vismia laurentii*, see: Kerharo (1974 ▶); Macfoy & Sama (1983 ▶). For other classes of natural products isolated from *Vismia* species, see: Simmonds *et al.* (1985 ▶); Nagem & de Oliveira (1997 ▶); Seo *et al.* (2000 ▶); Nguemeving *et al.* (2006 ▶). For related structures, see: Noungoue *et al.* (2008 ▶). 
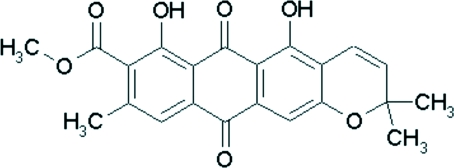


## Experimental

### 

#### Crystal data

C_22_H_18_O_7_
*M* *_r_* = 394.36
Triclinic, 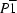

*a* = 6.9234 (4) Å
*b* = 16.0765 (9) Å
*c* = 17.5304 (9) Å
α = 108.746 (2)°
β = 98.725 (3)°
γ = 94.147 (2)°
*V* = 1810.97 (17) Å^3^
*Z* = 4
Mo *K*α radiation
μ = 0.11 mm^−1^
*T* = 173 (2) K
0.30 × 0.20 × 0.15 mm

#### Data collection

Nonius KappaCCD diffractometer
Absorption correction: none
17235 measured reflections
8260 independent reflections
4538 reflections with *I* > 2σ(*I*)
*R*_int_ = 0.062

#### Refinement

*R*[*F* ^2^ > 2σ(*F* ^2^)] = 0.072
*wR*(*F* ^2^) = 0.171
*S* = 1.02
8260 reflections
547 parameters
H atoms treated by a mixture of independent and constrained refinement
Δρ_max_ = 0.28 e Å^−3^
Δρ_min_ = −0.25 e Å^−3^

### 

Data collection: *COLLECT* (Hooft, 1998 ▶); cell refinement: *DENZO* (Otwinowski & Minor, 1997 ▶) and *COLLECT*; data reduction: *DENZO* and *COLLECT*; program(s) used to solve structure: *SHELXS97* (Sheldrick, 2008 ▶); program(s) used to refine structure: *SHELXL97* (Sheldrick, 2008 ▶); molecular graphics: *PLATON* (Spek, 2003 ▶); software used to prepare material for publication: *SHELXL97*.

## Supplementary Material

Crystal structure: contains datablocks global, I. DOI: 10.1107/S160053680803794X/hk2545sup1.cif

Structure factors: contains datablocks I. DOI: 10.1107/S160053680803794X/hk2545Isup2.hkl

Additional supplementary materials:  crystallographic information; 3D view; checkCIF report

## Figures and Tables

**Table 1:** Hydrogen-bond geometry (Å, °)

*D*—H⋯*A*	*D*—H	H⋯*A*	*D*⋯*A*	*D*—H⋯*A*
O4—H4*O*⋯O3	0.91 (3)	1.72 (3)	2.567 (2)	152 (3)
O11—H11O⋯O10	0.88 (3)	1.75 (4)	2.568 (2)	153 (3)
O9—H9*O*⋯O10	0.92 (3)	1.72 (3)	2.558 (2)	150 (3)
O2—H2O⋯O3	0.88 (3)	1.77 (3)	2.562 (2)	148 (3)
O2—H20⋯O9^i^	0.88 (3)	2.31 (3)	2.654 (2)	103 (2)
C34—H34⋯O7^ii^	0.95	2.59	3.441 (2)	150
C44—H44*B*⋯O7^ii^	0.98	2.51	3.423 (2)	155
C44—H44*C*⋯O8^ii^	0.98	2.58	3.419 (2)	144

Symmetry codes: (i) 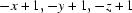; (ii) 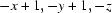.

**Fig. 1.**
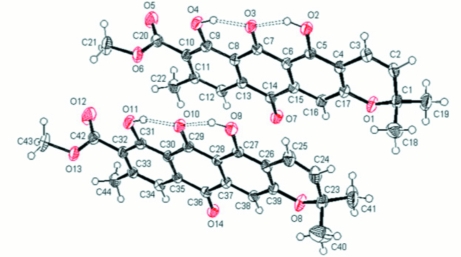
The molecular structure of the title molecule, with the atom-numbering scheme. Displacement ellipsoids are drawn at the 50% probability level.

## supplementary crystallographic information

### Comment

Anthraquinones are a class of natural products encompassing several hundreds of compounds. They are found in a large number of plant families particularly in Rubiaceae, Gesneriaceae, Polygonaceae, Guttiferae, fungi or lichen. Anthraquinones can be formed biosynthetically from shikimic acid, α-ketoglutarate and mevalonate or from acetate and malonate along the polyketide pathway (Birch *et al.*,  1965; Shibata & Ikekawa, 1963). Those naturally occurring compounds exhibit some interesting *in vivo* biological activities such as antimalarial, antileukemic, antibacterial (Adwankar & Chitnis, 1982; Sittie *et al.*,  1999; Rath *et al.*,  1995; Ismail *et al.*,  1997). Several Vismia species are known as sources of anthraquinones (Nagem & de Oliveira, 1997; Nguemeving *et al.*,  2006). They are used in traditional medicine as purgative, tonic or febrifugal agents and also for the treatment of skin diseases (Kerharo, 1974; Macfoy & Sama, 1983). Previous phytochemical investigations of Vismia species have revealed the presence of benzophenones, xanthones, triterpenoids and also anthraquinones (Simmonds *et al.*,  1985, Seo *et al.*, 2000). In a continuation of our search for bioactive compounds from Vismia laurentii, we have isolated from the EtOAc extract of the fruits 5 compounds comprising emodin, isoxanthorin, and three new ones laurentiquinones A, B(1) and C (Noungoue *et al.*, 2008). We reported herein the crystal structure of (1).

The asymmetric unit of the title compound contains two independent molecules, (Fig. 1). Rings B (C4-C6/C15-C17), C (C6-C8/C13-C15), D (C8-C13) and F (C26-C28/C37-C39), G (C28-C30/C35-C37), H (C30-C35) are, of course, planar and the dihedral angles between them are B/C = 1.11 (3)°, B/D = 2.86 (3)°, C/D = 1.75 (3)° and F/G = 1.43(39°, F/H = 1.59 (3)°, G/H = 1.57 (3)°. So, rings B, C, D and F, G, H are almost coplanar. Rings A (O1/C1-C4/C17) and E (O8/C23-C26/C39) adopt envelope conformations with C1 and C23 atoms displaced by 0.348 (3) Å and 0.192 (3) Å from the planes of the other rings atoms. The intramolecular O-H···O hydrogen bonds (Table 1) result in the formation of planar rings I (O3/O4/C7-C9/H4O), J (O2/O3/C5-C7/H2O) and K (O10/O11/C29-C31/H11O), L (O9/O10/C27-C29/H9O). They are also almost coplanar with the adjacent rings.

In the crystal structure, intermolecular O-H···O and C-H···O hydrogen bonds (Table 1) link the molecules, in which they may be effective in the stabilization of the structure. There also exist a π—π contact between G and H rings, Cg8···Cg7^i^ [symmetry code: (i) -x, 1 - y, -z, where Cg8 and Cg7 are the centroids of the rings H (C30-C35) and G (C28-C30/C35-C39) may further stabilize the structure, with centroid-centroid distance of 3.967 (3) Å.

### Experimental

The fruits of Vismia laurentii were collected from the bank of the Nyong river near Nkolmaka Lake (Endome) in Center Province, Cameroon on 17t h October 2004 by Mr. Nana Victor. A voucher specimen (No. 1882/SRFK) has been deposited in the National Herbarium, Yaounde, Cameroon. Dried fruits (0.988 kg) of V. laurentii were grounded and exhaustively extracted by maceration successively with hexane, ethyl acetate and methanol at room temperature. In each extraction 3x5 *L* of solvent were used for a period of 3x24 h and the extracts obtained were concentrated to dryness to give green (62.3 g), brown (43.6 g) and brown (22.1 g) crude viscous residues from hexane, EtOAc and MeOH extracts, respectively. The EtOAc extract (40 g) was subjected to flash column chromatography on silica gel 60 (0.063-0.200 mm, Merck, 500 g) as a stationary phase eluting with cyclohexane-EtOAc-MeOH mixtures of increasing polarity. Twenty-four fractions of 200 ml each were collected and grouped on the basis of TLC analysis to afford two main fractions A (11.7 g) and B (17.3 g). Fractions A and B were chromatographed on a silica gel column, using as eluent gradient mixtures of cyclohexane and EtOAc to yield laurentiquinone B (16 mg) in addition to other compounds. Orange-red crystals of the title compound were grown from a hexane-chloroform solution of laurentiquinone B.

### Refinement

H2O, H4O, H9O and H11O (for OH) were located in difference syntheses and refined isotropically [O-H = 0.88 (3)-0.92 (3) Å and U_iso_(H) = 0.069 (10)-0.092 (13) Å^2^]. The remaining H atoms were positioned geometrically, with C-H = 0.95 and 0.98 Å for aromatic and methyl H, respectively, and constrained to ride on their parent atoms with U_iso_(H) = xU_eq_(C), where x = 1.5 for methyl H and x = 1.2 for aromatic H atoms.

### Crystal data

**Table:**

C_22_H_18_O_7_	*Z* = 4
*M**_r_* = 394.36	*F*_000_ = 824
Triclinic, *P*1	*D*_x_ = 1.446 Mg m^−^^3^
Hall symbol: -P 1	Mo *K*α radiation λ = 0.71073 Å
*a* = 6.9234 (4) Å	Cell parameters from 8986 reflections
*b* = 16.0765 (9) Å	θ = 1.0–27.5º
*c* = 17.5304 (9) Å	µ = 0.11 mm^−^^1^
α = 108.746 (2)º	*T* = 173 (2) K
β = 98.725 (3)º	Plate, orange
γ = 94.147 (2)º	0.30 × 0.20 × 0.15 mm
*V* = 1810.97 (17) Å^3^	

### Data collection

**Table:**

Nonius KappaCCD diffractometer	4538 reflections with *I* > 2σ(*I*)
Radiation source: fine-focus sealed tube	*R*_int_ = 0.062
Monochromator: graphite	θ_max_ = 27.6º
*T* = 173(2) K	θ_min_ = 1.3º
φ and ω scans	*h* = −8→6
Absorption correction: none	*k* = −20→20
17235 measured reflections	*l* = −22→22
8260 independent reflections	

### Refinement

**Table:**

Refinement on *F*^2^	Secondary atom site location: difference Fourier map
Least-squares matrix: full	Hydrogen site location: inferred from neighbouring sites
*R*[*F*^2^ > 2σ(*F*^2^)] = 0.072	H atoms treated by a mixture of independent and constrained refinement
*wR*(*F*^2^) = 0.171	*w* = 1/[σ^2^(*F*_o_^2^) + (0.0685*P*)^2^ + 0.3328*P*] where *P* = (*F*_o_^2^ + 2*F*_c_^2^)/3
*S* = 1.02	(Δ/σ)_max_ < 0.001
8260 reflections	Δρ_max_ = 0.28 e Å^−^^3^
547 parameters	Δρ_min_ = −0.24 e Å^−^^3^
Primary atom site location: structure-invariant direct methods	Extinction correction: none

### Special details

**Table:**

Geometry. All e.s.d.'s (except the e.s.d. in the dihedral angle between two l.s. planes) are estimated using the full covariance matrix. The cell e.s.d.'s are taken into account individually in the estimation of e.s.d.'s in distances, angles and torsion angles; correlations between e.s.d.'s in cell parameters are only used when they are defined by crystal symmetry. An approximate (isotropic) treatment of cell e.s.d.'s is used for estimating e.s.d.'s involving l.s. planes.
Refinement. Refinement of *F*^2^ against ALL reflections. The weighted *R*-factor *wR* and goodness of fit *S* are based on *F*^2^, conventional *R*-factors *R* are based on *F*, with *F* set to zero for negative *F*^2^. The threshold expression of *F*^2^ > σ(*F*^2^) is used only for calculating *R*-factors(gt) *etc*. and is not relevant to the choice of reflections for refinement. *R*-factors based on *F*^2^ are statistically about twice as large as those based on *F*, and *R*- factors based on ALL data will be even larger.

### Fractional atomic coordinates and isotropic or equivalent isotropic displacement parameters (Å^2^)

**Table:**

	*x*	*y*	*z*	*U*_iso_*/*U*_eq_	
O1	0.9256 (3)	0.20606 (10)	0.34068 (10)	0.0353 (4)	
O2	0.7679 (3)	0.44308 (11)	0.55456 (9)	0.0297 (4)	
H2O	0.742 (4)	0.498 (2)	0.5634 (18)	0.069 (10)*	
O3	0.6830 (2)	0.58232 (10)	0.52267 (9)	0.0284 (4)	
O4	0.5973 (3)	0.72378 (11)	0.49464 (9)	0.0311 (4)	
H4O	0.614 (5)	0.682 (2)	0.5196 (19)	0.082 (11)*	
O5	0.6860 (3)	0.89104 (11)	0.42063 (12)	0.0494 (5)	
O6	0.3755 (3)	0.82417 (10)	0.38316 (10)	0.0371 (4)	
O7	0.8008 (3)	0.43662 (10)	0.21238 (9)	0.0335 (4)	
O8	0.3921 (3)	0.20125 (10)	0.08818 (10)	0.0375 (5)	
O9	0.2613 (3)	0.45070 (11)	0.29753 (9)	0.0310 (4)	
H9O	0.231 (5)	0.507 (2)	0.3026 (19)	0.078 (11)*	
O10	0.1911 (2)	0.58838 (10)	0.26044 (9)	0.0298 (4)	
O11	0.1139 (3)	0.72764 (11)	0.22628 (10)	0.0327 (4)	
H11O	0.128 (5)	0.689 (2)	0.252 (2)	0.092 (13)*	
O12	0.1994 (3)	0.88730 (12)	0.15164 (14)	0.0650 (7)	
O13	−0.1093 (3)	0.81995 (10)	0.10974 (10)	0.0352 (4)	
O14	0.2996 (2)	0.42633 (10)	−0.04866 (9)	0.0311 (4)	
C1	0.9107 (4)	0.14397 (15)	0.38645 (15)	0.0335 (6)	
C2	0.9295 (4)	0.19223 (15)	0.47656 (14)	0.0312 (6)	
H2	0.9744	0.1633	0.5141	0.037*	
C3	0.8851 (3)	0.27426 (15)	0.50542 (14)	0.0284 (6)	
H3	0.8830	0.3005	0.5621	0.034*	
C4	0.8398 (3)	0.32371 (14)	0.44995 (13)	0.0238 (5)	
C5	0.7869 (3)	0.40951 (14)	0.47550 (13)	0.0231 (5)	
C6	0.7611 (3)	0.45848 (14)	0.42189 (13)	0.0232 (5)	
C7	0.7075 (3)	0.54731 (14)	0.44998 (13)	0.0227 (5)	
C8	0.6849 (3)	0.59752 (14)	0.39322 (13)	0.0234 (5)	
C9	0.6330 (3)	0.68391 (14)	0.41885 (13)	0.0252 (5)	
C10	0.6215 (3)	0.73234 (14)	0.36560 (14)	0.0256 (5)	
C11	0.6576 (3)	0.69623 (15)	0.28607 (14)	0.0255 (5)	
C12	0.7058 (3)	0.61005 (15)	0.26013 (14)	0.0256 (5)	
H12	0.7294	0.5847	0.2060	0.031*	
C13	0.7199 (3)	0.56095 (14)	0.31249 (13)	0.0236 (5)	
C14	0.7733 (3)	0.46976 (14)	0.28247 (13)	0.0245 (5)	
C15	0.7928 (3)	0.41905 (14)	0.34075 (13)	0.0235 (5)	
C16	0.8462 (3)	0.33469 (14)	0.31475 (13)	0.0255 (5)	
H16	0.8686	0.3091	0.2604	0.031*	
C17	0.8669 (3)	0.28760 (14)	0.36917 (14)	0.0267 (5)	
C18	0.7102 (4)	0.08818 (18)	0.35335 (17)	0.0492 (8)	
H18A	0.6065	0.1265	0.3641	0.074*	
H18B	0.6984	0.0439	0.3805	0.074*	
H18C	0.6965	0.0582	0.2941	0.074*	
C19	1.0782 (4)	0.08948 (17)	0.36805 (17)	0.0479 (7)	
H19A	1.0675	0.0631	0.3086	0.072*	
H19B	1.0711	0.0424	0.3921	0.072*	
H19C	1.2043	0.1277	0.3916	0.072*	
C20	0.5689 (4)	0.82504 (16)	0.39418 (14)	0.0310 (6)	
C21	0.3033 (4)	0.90901 (17)	0.39716 (18)	0.0502 (8)	
H21A	0.3422	0.9449	0.4553	0.075*	
H21B	0.1592	0.8999	0.3818	0.075*	
H21C	0.3594	0.9397	0.3639	0.075*	
C22	0.6469 (4)	0.75002 (16)	0.22934 (15)	0.0346 (6)	
H22A	0.5282	0.7797	0.2322	0.052*	
H22B	0.6419	0.7107	0.1730	0.052*	
H22C	0.7637	0.7945	0.2460	0.052*	
C23	0.3687 (4)	0.14102 (15)	0.13466 (15)	0.0362 (6)	
C24	0.3587 (4)	0.18934 (17)	0.22190 (16)	0.0390 (7)	
H24	0.3704	0.1574	0.2591	0.047*	
C25	0.3347 (4)	0.27396 (16)	0.25069 (15)	0.0354 (6)	
H25	0.3259	0.3010	0.3066	0.042*	
C26	0.3219 (3)	0.32519 (15)	0.19542 (13)	0.0250 (5)	
C27	0.2816 (3)	0.41312 (15)	0.21919 (13)	0.0239 (5)	
C28	0.2650 (3)	0.46045 (14)	0.16364 (13)	0.0219 (5)	
C29	0.2162 (3)	0.54995 (14)	0.18846 (13)	0.0238 (5)	
C30	0.1976 (3)	0.59654 (14)	0.12902 (13)	0.0236 (5)	
C31	0.1486 (3)	0.68315 (15)	0.15087 (13)	0.0258 (5)	
C32	0.1365 (3)	0.72859 (14)	0.09460 (14)	0.0257 (5)	
C33	0.1732 (3)	0.68879 (15)	0.01611 (14)	0.0254 (5)	
C34	0.2184 (3)	0.60181 (15)	−0.00646 (13)	0.0255 (5)	
H34	0.2415	0.5737	−0.0603	0.031*	
C35	0.2301 (3)	0.55620 (14)	0.04830 (13)	0.0229 (5)	
C36	0.2777 (3)	0.46361 (14)	0.02161 (13)	0.0234 (5)	
C37	0.2963 (3)	0.41710 (14)	0.08309 (13)	0.0223 (5)	
C38	0.3410 (3)	0.33106 (14)	0.05955 (14)	0.0249 (5)	
H38	0.3651	0.3032	0.0058	0.030*	
C39	0.3502 (3)	0.28573 (14)	0.11525 (14)	0.0254 (5)	
C40	0.1808 (5)	0.0794 (2)	0.0916 (2)	0.0791 (12)	
H40A	0.0681	0.1130	0.0972	0.119*	
H40B	0.1666	0.0331	0.1162	0.119*	
H40C	0.1857	0.0522	0.0333	0.119*	
C41	0.5481 (5)	0.0925 (2)	0.12807 (19)	0.0678 (10)	
H41A	0.5594	0.0673	0.0703	0.102*	
H41B	0.5347	0.0448	0.1512	0.102*	
H41C	0.6665	0.1340	0.1585	0.102*	
C42	0.0842 (4)	0.82113 (16)	0.12240 (14)	0.0315 (6)	
C43	−0.1864 (4)	0.90363 (17)	0.13526 (18)	0.0479 (8)	
H43A	−0.1394	0.9338	0.1942	0.072*	
H43B	−0.3308	0.8932	0.1239	0.072*	
H43C	−0.1415	0.9407	0.1050	0.072*	
C44	0.1659 (4)	0.73895 (16)	−0.04345 (15)	0.0332 (6)	
H44A	0.0585	0.7756	−0.0370	0.050*	
H44B	0.1433	0.6969	−0.0996	0.050*	
H44C	0.2913	0.7769	−0.0325	0.050*	

### Atomic displacement parameters (Å^2^)

**Table:**

	*U*^11^	*U*^22^	*U*^33^	*U*^12^	*U*^13^	*U*^23^
O1	0.0528 (12)	0.0233 (9)	0.0364 (10)	0.0154 (8)	0.0186 (9)	0.0120 (8)
O2	0.0440 (11)	0.0264 (10)	0.0218 (9)	0.0105 (8)	0.0097 (8)	0.0092 (7)
O3	0.0379 (10)	0.0267 (9)	0.0224 (8)	0.0108 (7)	0.0100 (8)	0.0071 (7)
O4	0.0419 (11)	0.0255 (9)	0.0272 (9)	0.0099 (8)	0.0115 (8)	0.0071 (8)
O5	0.0432 (12)	0.0245 (10)	0.0737 (14)	0.0003 (9)	−0.0003 (10)	0.0132 (9)
O6	0.0349 (11)	0.0276 (9)	0.0510 (11)	0.0134 (8)	0.0081 (9)	0.0140 (8)
O7	0.0500 (12)	0.0301 (9)	0.0241 (9)	0.0116 (8)	0.0146 (8)	0.0091 (7)
O8	0.0563 (12)	0.0222 (9)	0.0423 (10)	0.0125 (8)	0.0219 (9)	0.0147 (8)
O9	0.0422 (11)	0.0325 (10)	0.0221 (9)	0.0114 (8)	0.0089 (8)	0.0116 (8)
O10	0.0404 (11)	0.0280 (9)	0.0232 (8)	0.0100 (8)	0.0106 (8)	0.0084 (7)
O11	0.0472 (12)	0.0268 (9)	0.0256 (9)	0.0156 (8)	0.0108 (8)	0.0067 (8)
O12	0.0509 (14)	0.0246 (11)	0.1069 (18)	−0.0017 (10)	0.0025 (13)	0.0118 (11)
O13	0.0359 (11)	0.0254 (9)	0.0455 (10)	0.0130 (8)	0.0104 (9)	0.0103 (8)
O14	0.0425 (11)	0.0295 (9)	0.0234 (9)	0.0103 (8)	0.0106 (8)	0.0084 (7)
C1	0.0412 (16)	0.0256 (13)	0.0380 (14)	0.0107 (11)	0.0058 (12)	0.0159 (11)
C2	0.0339 (15)	0.0311 (14)	0.0340 (14)	0.0103 (11)	0.0055 (12)	0.0174 (11)
C3	0.0268 (14)	0.0310 (14)	0.0315 (13)	0.0065 (11)	0.0060 (11)	0.0150 (11)
C4	0.0233 (13)	0.0229 (12)	0.0270 (12)	0.0034 (10)	0.0065 (10)	0.0099 (10)
C5	0.0238 (13)	0.0239 (12)	0.0212 (12)	0.0032 (10)	0.0056 (10)	0.0065 (10)
C6	0.0236 (13)	0.0227 (12)	0.0237 (12)	0.0030 (10)	0.0061 (10)	0.0074 (10)
C7	0.0208 (12)	0.0227 (12)	0.0245 (12)	0.0018 (10)	0.0050 (10)	0.0076 (10)
C8	0.0232 (13)	0.0221 (12)	0.0264 (12)	0.0045 (10)	0.0063 (10)	0.0091 (10)
C9	0.0238 (13)	0.0222 (12)	0.0284 (12)	0.0005 (10)	0.0061 (11)	0.0067 (10)
C10	0.0231 (13)	0.0207 (12)	0.0321 (13)	0.0025 (10)	0.0046 (11)	0.0083 (10)
C11	0.0237 (13)	0.0261 (13)	0.0301 (13)	0.0030 (10)	0.0057 (11)	0.0139 (10)
C12	0.0243 (13)	0.0279 (13)	0.0266 (12)	0.0041 (10)	0.0067 (10)	0.0108 (10)
C13	0.0206 (13)	0.0259 (12)	0.0260 (12)	0.0037 (10)	0.0072 (10)	0.0096 (10)
C14	0.0246 (13)	0.0245 (13)	0.0233 (12)	0.0024 (10)	0.0065 (10)	0.0059 (10)
C15	0.0243 (13)	0.0226 (12)	0.0248 (12)	0.0023 (10)	0.0071 (10)	0.0088 (10)
C16	0.0299 (14)	0.0240 (12)	0.0241 (12)	0.0046 (10)	0.0095 (11)	0.0080 (10)
C17	0.0281 (14)	0.0218 (12)	0.0310 (13)	0.0053 (10)	0.0080 (11)	0.0084 (10)
C18	0.055 (2)	0.0458 (17)	0.0454 (17)	−0.0039 (15)	−0.0032 (15)	0.0207 (14)
C19	0.063 (2)	0.0366 (16)	0.0481 (17)	0.0264 (14)	0.0138 (15)	0.0131 (13)
C20	0.0345 (15)	0.0311 (14)	0.0287 (13)	0.0050 (12)	0.0037 (12)	0.0129 (11)
C21	0.053 (2)	0.0341 (16)	0.071 (2)	0.0217 (14)	0.0164 (16)	0.0229 (15)
C22	0.0397 (16)	0.0339 (14)	0.0365 (14)	0.0070 (12)	0.0093 (12)	0.0188 (12)
C23	0.0480 (18)	0.0236 (13)	0.0411 (15)	0.0041 (12)	0.0049 (13)	0.0180 (12)
C24	0.0464 (17)	0.0383 (16)	0.0457 (16)	0.0127 (13)	0.0159 (14)	0.0275 (13)
C25	0.0425 (16)	0.0369 (15)	0.0356 (14)	0.0122 (12)	0.0154 (13)	0.0190 (12)
C26	0.0218 (13)	0.0284 (13)	0.0284 (12)	0.0043 (10)	0.0054 (10)	0.0137 (10)
C27	0.0236 (13)	0.0281 (13)	0.0205 (11)	0.0045 (10)	0.0050 (10)	0.0084 (10)
C28	0.0212 (13)	0.0223 (12)	0.0217 (11)	0.0024 (10)	0.0024 (10)	0.0073 (10)
C29	0.0208 (13)	0.0262 (13)	0.0232 (12)	0.0024 (10)	0.0043 (10)	0.0067 (10)
C30	0.0230 (13)	0.0228 (12)	0.0242 (12)	0.0017 (10)	0.0039 (10)	0.0075 (10)
C31	0.0249 (14)	0.0269 (13)	0.0240 (12)	0.0034 (10)	0.0039 (10)	0.0067 (10)
C32	0.0245 (13)	0.0209 (12)	0.0317 (13)	0.0031 (10)	0.0038 (11)	0.0095 (10)
C33	0.0222 (13)	0.0268 (13)	0.0285 (12)	0.0017 (10)	0.0031 (10)	0.0121 (10)
C34	0.0243 (13)	0.0293 (13)	0.0251 (12)	0.0041 (10)	0.0069 (10)	0.0112 (10)
C35	0.0186 (12)	0.0249 (12)	0.0246 (12)	0.0024 (10)	0.0041 (10)	0.0075 (10)
C36	0.0199 (12)	0.0273 (13)	0.0233 (12)	0.0022 (10)	0.0051 (10)	0.0087 (10)
C37	0.0211 (12)	0.0231 (12)	0.0235 (12)	0.0014 (10)	0.0064 (10)	0.0081 (10)
C38	0.0241 (13)	0.0238 (12)	0.0254 (12)	0.0016 (10)	0.0049 (10)	0.0067 (10)
C39	0.0229 (13)	0.0209 (12)	0.0326 (13)	0.0039 (10)	0.0067 (11)	0.0084 (10)
C40	0.085 (3)	0.073 (2)	0.075 (2)	−0.041 (2)	−0.019 (2)	0.045 (2)
C41	0.098 (3)	0.070 (2)	0.0523 (19)	0.059 (2)	0.0272 (19)	0.0272 (17)
C42	0.0387 (16)	0.0264 (14)	0.0319 (14)	0.0052 (12)	0.0070 (12)	0.0126 (11)
C43	0.061 (2)	0.0297 (15)	0.0617 (19)	0.0253 (14)	0.0266 (16)	0.0164 (13)
C44	0.0368 (15)	0.0319 (14)	0.0375 (14)	0.0086 (11)	0.0103 (12)	0.0185 (12)

### Geometric parameters (Å, °)

**Table:**

O2—H2O	0.88 (3)	C22—H22B	0.9800
O4—H4O	0.91 (3)	C22—H22C	0.9800
O9—H9O	0.92 (3)	C23—O8	1.467 (3)
O11—H11O	0.88 (3)	C23—C24	1.491 (3)
C1—O1	1.475 (3)	C23—C40	1.509 (4)
C1—C2	1.500 (3)	C23—C41	1.514 (4)
C1—C19	1.515 (3)	C24—C25	1.323 (3)
C1—C18	1.520 (3)	C24—H24	0.9500
C2—C3	1.329 (3)	C25—C26	1.457 (3)
C2—H2	0.9500	C25—H25	0.9500
C3—C4	1.455 (3)	C26—C39	1.395 (3)
C3—H3	0.9500	C26—C27	1.401 (3)
C4—C17	1.395 (3)	C27—O9	1.345 (2)
C4—C5	1.401 (3)	C27—C28	1.413 (3)
C5—O2	1.347 (2)	C28—C37	1.417 (3)
C5—C6	1.407 (3)	C28—C29	1.444 (3)
C6—C15	1.417 (3)	C29—O10	1.259 (2)
C6—C7	1.449 (3)	C29—C30	1.462 (3)
C7—O3	1.260 (2)	C30—C31	1.402 (3)
C7—C8	1.467 (3)	C30—C35	1.415 (3)
C8—C9	1.407 (3)	C31—O11	1.353 (3)
C8—C13	1.413 (3)	C31—C32	1.400 (3)
C9—O4	1.346 (3)	C32—C33	1.390 (3)
C9—C10	1.392 (3)	C32—C42	1.499 (3)
C10—C11	1.396 (3)	C33—C34	1.398 (3)
C10—C20	1.503 (3)	C33—C44	1.508 (3)
C11—C12	1.395 (3)	C34—C35	1.380 (3)
C11—C22	1.511 (3)	C34—H34	0.9500
C12—C13	1.387 (3)	C35—C36	1.488 (3)
C12—H12	0.9500	C36—O14	1.223 (2)
C13—C14	1.485 (3)	C36—C37	1.492 (3)
C14—O7	1.223 (2)	C37—C38	1.383 (3)
C14—C15	1.495 (3)	C38—C39	1.391 (3)
C15—C16	1.382 (3)	C38—H38	0.9500
C16—C17	1.394 (3)	C39—O8	1.356 (3)
C16—H16	0.9500	C40—H40A	0.9800
C17—O1	1.360 (3)	C40—H40B	0.9800
C18—H18A	0.9800	C40—H40C	0.9800
C18—H18B	0.9800	C41—H41A	0.9800
C18—H18C	0.9800	C41—H41B	0.9800
C19—H19A	0.9800	C41—H41C	0.9800
C19—H19B	0.9800	C42—O12	1.197 (3)
C19—H19C	0.9800	C42—O13	1.322 (3)
C20—O5	1.201 (3)	C43—O13	1.444 (3)
C20—O6	1.322 (3)	C43—H43A	0.9800
C21—O6	1.446 (3)	C43—H43B	0.9800
C21—H21A	0.9800	C43—H43C	0.9800
C21—H21B	0.9800	C44—H44A	0.9800
C21—H21C	0.9800	C44—H44B	0.9800
C22—H22A	0.9800	C44—H44C	0.9800
			
C17—O1—C1	120.10 (17)	H22A—C22—H22B	109.5
C5—O2—H2O	107.1 (19)	C11—C22—H22C	109.5
C9—O4—H4O	104 (2)	H22A—C22—H22C	109.5
C20—O6—C21	116.6 (2)	H22B—C22—H22C	109.5
C39—O8—C23	121.75 (17)	O8—C23—C24	112.24 (19)
C27—O9—H9O	106.2 (19)	O8—C23—C40	106.2 (2)
C31—O11—H11O	103 (2)	C24—C23—C40	110.8 (2)
C42—O13—C43	117.9 (2)	O8—C23—C41	104.3 (2)
O1—C1—C2	111.49 (18)	C24—C23—C41	111.3 (2)
O1—C1—C19	104.25 (18)	C40—C23—C41	111.8 (3)
C2—C1—C19	111.7 (2)	C25—C24—C23	124.1 (2)
O1—C1—C18	107.4 (2)	C25—C24—H24	118.0
C2—C1—C18	109.5 (2)	C23—C24—H24	118.0
C19—C1—C18	112.2 (2)	C24—C25—C26	119.1 (2)
C3—C2—C1	122.0 (2)	C24—C25—H25	120.4
C3—C2—H2	119.0	C26—C25—H25	120.4
C1—C2—H2	119.0	C39—C26—C27	118.0 (2)
C2—C3—C4	119.8 (2)	C39—C26—C25	119.2 (2)
C2—C3—H3	120.1	C27—C26—C25	122.8 (2)
C4—C3—H3	120.1	O9—C27—C26	116.96 (19)
C17—C4—C5	118.0 (2)	O9—C27—C28	121.5 (2)
C17—C4—C3	118.7 (2)	C26—C27—C28	121.56 (19)
C5—C4—C3	123.07 (19)	C27—C28—C37	117.9 (2)
O2—C5—C4	116.25 (19)	C27—C28—C29	120.72 (19)
O2—C5—C6	122.1 (2)	C37—C28—C29	121.40 (19)
C4—C5—C6	121.58 (19)	O10—C29—C28	121.0 (2)
C5—C6—C15	118.1 (2)	O10—C29—C30	119.8 (2)
C5—C6—C7	120.50 (19)	C28—C29—C30	119.24 (18)
C15—C6—C7	121.4 (2)	C31—C30—C35	118.1 (2)
O3—C7—C6	120.89 (19)	C31—C30—C29	120.71 (19)
O3—C7—C8	119.7 (2)	C35—C30—C29	121.2 (2)
C6—C7—C8	119.38 (19)	O11—C31—C32	116.6 (2)
C9—C8—C13	118.7 (2)	O11—C31—C30	122.8 (2)
C9—C8—C7	120.59 (19)	C32—C31—C30	120.61 (19)
C13—C8—C7	120.6 (2)	C33—C32—C31	120.7 (2)
O4—C9—C10	117.4 (2)	C33—C32—C42	121.7 (2)
O4—C9—C8	122.6 (2)	C31—C32—C42	117.63 (19)
C10—C9—C8	119.9 (2)	C32—C33—C34	118.9 (2)
C9—C10—C11	121.2 (2)	C32—C33—C44	120.5 (2)
C9—C10—C20	119.4 (2)	C34—C33—C44	120.6 (2)
C11—C10—C20	119.4 (2)	C35—C34—C33	121.0 (2)
C12—C11—C10	119.0 (2)	C35—C34—H34	119.5
C12—C11—C22	120.5 (2)	C33—C34—H34	119.5
C10—C11—C22	120.5 (2)	C34—C35—C30	120.7 (2)
C13—C12—C11	120.8 (2)	C34—C35—C36	119.22 (19)
C13—C12—H12	119.6	C30—C35—C36	120.04 (19)
C11—C12—H12	119.6	O14—C36—C35	121.2 (2)
C12—C13—C8	120.4 (2)	O14—C36—C37	120.8 (2)
C12—C13—C14	118.95 (19)	C35—C36—C37	117.98 (18)
C8—C13—C14	120.66 (19)	C38—C37—C28	121.0 (2)
O7—C14—C13	121.5 (2)	C38—C37—C36	118.88 (19)
O7—C14—C15	120.6 (2)	C28—C37—C36	120.08 (19)
C13—C14—C15	117.95 (18)	C37—C38—C39	119.4 (2)
C16—C15—C6	121.0 (2)	C37—C38—H38	120.3
C16—C15—C14	119.04 (19)	C39—C38—H38	120.3
C6—C15—C14	119.9 (2)	O8—C39—C38	116.58 (19)
C15—C16—C17	119.2 (2)	O8—C39—C26	121.3 (2)
C15—C16—H16	120.4	C38—C39—C26	122.1 (2)
C17—C16—H16	120.4	C23—C40—H40A	109.5
O1—C17—C16	116.38 (19)	C23—C40—H40B	109.5
O1—C17—C4	121.5 (2)	H40A—C40—H40B	109.5
C16—C17—C4	122.0 (2)	C23—C40—H40C	109.5
C1—C18—H18A	109.5	H40A—C40—H40C	109.5
C1—C18—H18B	109.5	H40B—C40—H40C	109.5
H18A—C18—H18B	109.5	C23—C41—H41A	109.5
C1—C18—H18C	109.5	C23—C41—H41B	109.5
H18A—C18—H18C	109.5	H41A—C41—H41B	109.5
H18B—C18—H18C	109.5	C23—C41—H41C	109.5
C1—C19—H19A	109.5	H41A—C41—H41C	109.5
C1—C19—H19B	109.5	H41B—C41—H41C	109.5
H19A—C19—H19B	109.5	O12—C42—O13	124.1 (2)
C1—C19—H19C	109.5	O12—C42—C32	125.5 (2)
H19A—C19—H19C	109.5	O13—C42—C32	110.3 (2)
H19B—C19—H19C	109.5	O13—C43—H43A	109.5
O5—C20—O6	124.5 (2)	O13—C43—H43B	109.5
O5—C20—C10	124.8 (2)	H43A—C43—H43B	109.5
O6—C20—C10	110.6 (2)	O13—C43—H43C	109.5
O6—C21—H21A	109.5	H43A—C43—H43C	109.5
O6—C21—H21B	109.5	H43B—C43—H43C	109.5
H21A—C21—H21B	109.5	C33—C44—H44A	109.5
O6—C21—H21C	109.5	C33—C44—H44B	109.5
H21A—C21—H21C	109.5	H44A—C44—H44B	109.5
H21B—C21—H21C	109.5	C33—C44—H44C	109.5
C11—C22—H22A	109.5	H44A—C44—H44C	109.5
C11—C22—H22B	109.5	H44B—C44—H44C	109.5
			
O1—C1—C2—C3	24.9 (3)	C25—C26—C27—O9	2.3 (3)
C19—C1—C2—C3	141.1 (2)	C39—C26—C27—C28	1.6 (3)
C18—C1—C2—C3	−93.9 (3)	C25—C26—C27—C28	−178.3 (2)
C1—C2—C3—C4	−7.6 (4)	O9—C27—C28—C37	177.5 (2)
C2—C3—C4—C17	−7.6 (3)	C26—C27—C28—C37	−1.9 (3)
C2—C3—C4—C5	178.3 (2)	O9—C27—C28—C29	−2.9 (3)
C17—C4—C5—O2	−178.06 (19)	C26—C27—C28—C29	177.7 (2)
C3—C4—C5—O2	−3.9 (3)	C27—C28—C29—O10	1.4 (3)
C17—C4—C5—C6	0.1 (3)	C37—C28—C29—O10	−179.1 (2)
C3—C4—C5—C6	174.2 (2)	C27—C28—C29—C30	−179.2 (2)
O2—C5—C6—C15	177.2 (2)	C37—C28—C29—C30	0.4 (3)
C4—C5—C6—C15	−0.9 (3)	O10—C29—C30—C31	−1.2 (3)
O2—C5—C6—C7	−1.6 (3)	C28—C29—C30—C31	179.4 (2)
C4—C5—C6—C7	−179.6 (2)	O10—C29—C30—C35	178.2 (2)
C5—C6—C7—O3	0.0 (3)	C28—C29—C30—C35	−1.2 (3)
C15—C6—C7—O3	−178.8 (2)	C35—C30—C31—O11	179.8 (2)
C5—C6—C7—C8	178.9 (2)	C29—C30—C31—O11	−0.8 (3)
C15—C6—C7—C8	0.2 (3)	C35—C30—C31—C32	−1.3 (3)
O3—C7—C8—C9	−1.1 (3)	C29—C30—C31—C32	178.1 (2)
C6—C7—C8—C9	180.0 (2)	O11—C31—C32—C33	178.9 (2)
O3—C7—C8—C13	177.3 (2)	C30—C31—C32—C33	−0.1 (3)
C6—C7—C8—C13	−1.6 (3)	O11—C31—C32—C42	−0.8 (3)
C13—C8—C9—O4	−179.94 (19)	C30—C31—C32—C42	−179.7 (2)
C7—C8—C9—O4	−1.6 (3)	C31—C32—C33—C34	1.3 (3)
C13—C8—C9—C10	−1.5 (3)	C42—C32—C33—C34	−179.1 (2)
C7—C8—C9—C10	176.9 (2)	C31—C32—C33—C44	−178.2 (2)
O4—C9—C10—C11	179.6 (2)	C42—C32—C33—C44	1.4 (3)
C8—C9—C10—C11	1.1 (3)	C32—C33—C34—C35	−1.1 (3)
O4—C9—C10—C20	−0.5 (3)	C44—C33—C34—C35	178.4 (2)
C8—C9—C10—C20	−179.1 (2)	C33—C34—C35—C30	−0.3 (3)
C9—C10—C11—C12	0.0 (3)	C33—C34—C35—C36	179.7 (2)
C20—C10—C11—C12	−179.9 (2)	C31—C30—C35—C34	1.5 (3)
C9—C10—C11—C22	−179.3 (2)	C29—C30—C35—C34	−177.9 (2)
C20—C10—C11—C22	0.9 (3)	C31—C30—C35—C36	−178.5 (2)
C10—C11—C12—C13	−0.6 (3)	C29—C30—C35—C36	2.1 (3)
C22—C11—C12—C13	178.6 (2)	C34—C35—C36—O14	−2.6 (3)
C11—C12—C13—C8	0.2 (3)	C30—C35—C36—O14	177.4 (2)
C11—C12—C13—C14	−179.5 (2)	C34—C35—C36—C37	177.9 (2)
C9—C8—C13—C12	0.9 (3)	C30—C35—C36—C37	−2.1 (3)
C7—C8—C13—C12	−177.5 (2)	C27—C28—C37—C38	0.3 (3)
C9—C8—C13—C14	−179.4 (2)	C29—C28—C37—C38	−179.3 (2)
C7—C8—C13—C14	2.2 (3)	C27—C28—C37—C36	179.1 (2)
C12—C13—C14—O7	−1.5 (3)	C29—C28—C37—C36	−0.5 (3)
C8—C13—C14—O7	178.8 (2)	O14—C36—C37—C38	0.6 (3)
C12—C13—C14—C15	178.4 (2)	C35—C36—C37—C38	−179.8 (2)
C8—C13—C14—C15	−1.3 (3)	O14—C36—C37—C28	−178.2 (2)
C5—C6—C15—C16	0.4 (3)	C35—C36—C37—C28	1.3 (3)
C7—C6—C15—C16	179.2 (2)	C28—C37—C38—C39	1.6 (3)
C5—C6—C15—C14	−178.1 (2)	C36—C37—C38—C39	−177.2 (2)
C7—C6—C15—C14	0.7 (3)	C37—C38—C39—O8	179.2 (2)
O7—C14—C15—C16	1.2 (3)	C37—C38—C39—C26	−2.0 (3)
C13—C14—C15—C16	−178.7 (2)	C27—C26—C39—O8	179.2 (2)
O7—C14—C15—C6	179.8 (2)	C25—C26—C39—O8	−0.9 (3)
C13—C14—C15—C6	−0.2 (3)	C27—C26—C39—C38	0.5 (3)
C6—C15—C16—C17	0.7 (3)	C25—C26—C39—C38	−179.7 (2)
C14—C15—C16—C17	179.2 (2)	C33—C32—C42—O12	−86.8 (3)
C15—C16—C17—O1	−178.1 (2)	C31—C32—C42—O12	92.8 (3)
C15—C16—C17—C4	−1.5 (4)	C33—C32—C42—O13	92.9 (3)
C5—C4—C17—O1	177.5 (2)	C31—C32—C42—O13	−87.5 (3)
C3—C4—C17—O1	3.1 (3)	C16—C17—O1—C1	−166.9 (2)
C5—C4—C17—C16	1.1 (3)	C4—C17—O1—C1	16.5 (3)
C3—C4—C17—C16	−173.3 (2)	C2—C1—O1—C17	−29.0 (3)
C9—C10—C20—O5	96.9 (3)	C19—C1—O1—C17	−149.8 (2)
C11—C10—C20—O5	−83.2 (3)	C18—C1—O1—C17	91.0 (3)
C9—C10—C20—O6	−85.6 (3)	O5—C20—O6—C21	5.9 (4)
C11—C10—C20—O6	94.3 (2)	C10—C20—O6—C21	−171.6 (2)
O8—C23—C24—C25	12.5 (4)	C38—C39—O8—C23	−168.0 (2)
C40—C23—C24—C25	−106.0 (3)	C26—C39—O8—C23	13.3 (3)
C41—C23—C24—C25	129.0 (3)	C24—C23—O8—C39	−18.1 (3)
C23—C24—C25—C26	−1.7 (4)	C40—C23—O8—C39	103.1 (3)
C24—C25—C26—C39	−4.8 (4)	C41—C23—O8—C39	−138.7 (2)
C24—C25—C26—C27	175.0 (2)	O12—C42—O13—C43	−2.1 (4)
C39—C26—C27—O9	−177.88 (19)	C32—C42—O13—C43	178.22 (19)

### Hydrogen-bond geometry (Å, °)

**Table:**

*D*—H···*A*	*D*—H	H···*A*	*D*···*A*	*D*—H···*A*
O4—H4O···O3	0.91 (3)	1.72 (3)	2.567 (2)	152 (3)
O11—H11O···O10	0.88 (3)	1.75 (4)	2.568 (2)	153 (3)
O9—H9O···O10	0.92 (3)	1.72 (3)	2.558 (2)	150 (3)
O2—H2O···O3	0.88 (3)	1.77 (3)	2.562 (2)	148 (3)
O2—H20···O9^i^	0.88 (3)	2.31 (3)	2.654 (2)	103 (2)
C34—H34···O7^ii^	0.95	2.59	3.441 (2)	150
C44—H44B···O7^ii^	0.98	2.51	3.423 (2)	155
C44—H44C···O8^ii^	0.98	2.58	3.419 (2)	144

Symmetry codes: (i) −*x*+1, −*y*+1, −*z*+1; (ii) −*x*+1, −*y*+1, −*z*.
